# A systematic method of estimation of alongshore windstress and Ekman transport associated with coastal upwelling

**DOI:** 10.1016/j.mex.2023.102186

**Published:** 2023-04-15

**Authors:** Sthitapragya Ray, Debadatta Swain

**Affiliations:** School of Earth, Ocean and Climate Sciences, Indian Institute of Technology Bhubaneswar, Argul, Jatni 752050, India

**Keywords:** Coastal upwelling, Alongshore windstress, Coastal productivity, Coastal hydrography, Alongshore Windstress and Ekman Transport Estimation

## Abstract

A standard method for estimating alongshore windstress (AWS) and related cross-shore Ekman transport (ET) is proposed. In the absence of standard methodologies for estimating coastal angles required for AWS estimation, an estimation is typically derived on a case-to-case basis, often approximating entire coastlines with a single line segment. A novel standard parametric method for estimating this coastal angle is developed in this manuscript consisting:

• Computation of Coastal Angle from Global Self-consistent Hierarchical High-resolution Shoreline (GSHHS) coastline data through line simplification
• Estimation of Windstress components at coastal points from Copernicus Marine Environment Monitoring Service (CMEMS) wind data
• Estimation of AWS and associated Ekman Transport

The method developed was demonstrated for the eastern and western coasts of India. While, a single line approximation was adequate for representing the west coast of India (a nearly linear coastline), an ET variation greater than 20% was introduced by oversimplification of the coastline for the east coast, which has significant curvature. This demonstrated the relevance of our systematic approach for coastline simplification, emphasizing the significance of accurate determination of coastal angles for computing ET simultaneously.

## Graphical abstract

**Figure:**
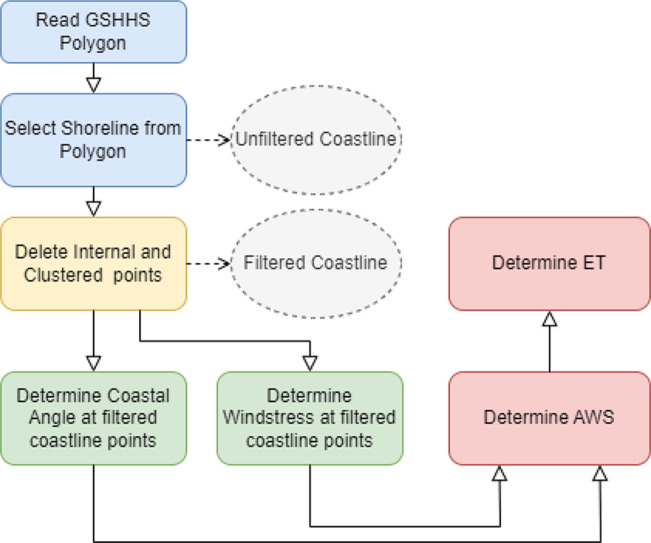

Specifications table

**Table:** 

Subject area:	Earth and Planetary Sciences
More specific subject area:	*Physical Oceanography*
Name of your method:	*Alongshore Windstress and Ekman Transport Estimation*
Name and reference of original method:	R. Varela, I. Álvarez, F. Santos, M. deCastro, M. Gómez-Gesteira, Has upwelling strengthened along worldwide coasts over 1982–2010?, Sci Rep. 5 (2015) 10,016. 10.1038/srep10016.
Resource availability:	https://data.marine.copernicus.eu/product/WIND_GLO_PHY_L4_MY_012_006/description https://www.soest.hawaii.edu/pwessel/gshhg/

## Method details

**Fig. 1:**
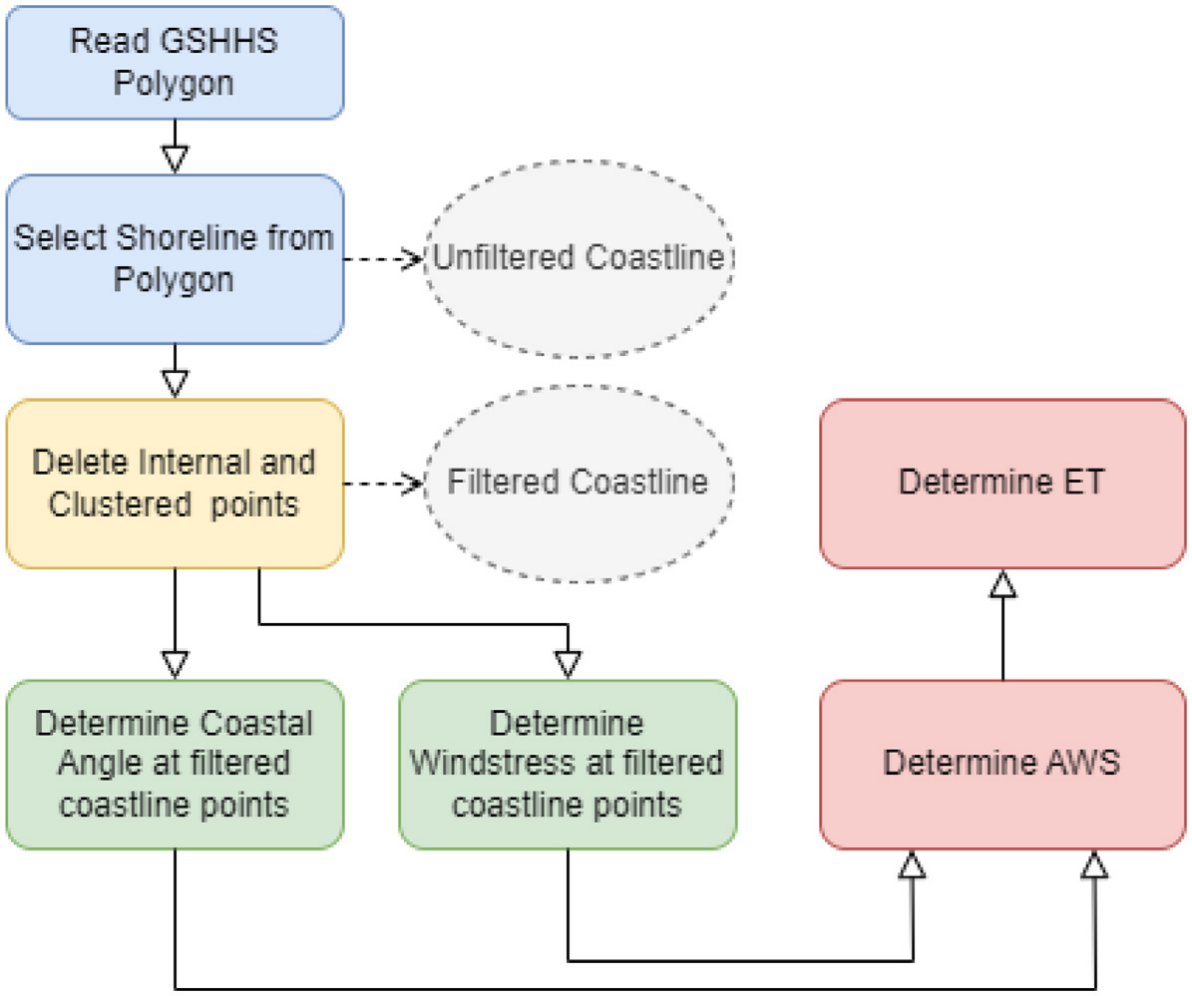
Outline of process.

Broadly, the method developed in this study consisted of 3 steps: estimation of coastal angle, estimation of windstress components, and computation of ET. An outline of this process is presented in Fig. 1, and details on each step are presented in the following subsections.

### Data used

The coastal angles are computed from the Global Self-consistent Hierarchical High-resolution Shoreline (GSHHS) database [12] version 2.3.7 (released on 15th June 2017), which integrates several open source shoreline datasets (particularly the World Vector Shoreline dataset [13]). This shoreline data is available as polygons in five resolutions (full (F), high (H), intermediate (I), low (L), and crude (C) resolution), with each having approximately 80% reduction in size and quality compared to the preceding one. The Douglas-Peucker (DP) line simplification algorithm [14] is used successively to lower the resolution of the shoreline polygons with increasing tolerance parameter values of 0.2 km (H), 1 km (I), 5 km (L), and 25 km (C), for example [12]. Further, the shorelines are classified into six hierarchical levels: Level 1 (land-ocean boundaries), Level 2 (Land-lake boundaries), Level 3 (boundaries between lakes and islands in lakes), Level 4 (boundaries between islands and ponds within islands), Level 5 (Antarctic ice-ocean boundaries), and Level 6 (boundaries between Antarctic grounding-line and ocean). The present method uses low-resolution L1 coastline polygons. Additionally, 6-hourly windstress data on a 0.25° × 0.25° grid from the Institut Français de Recherche pour l'Exploitation de la Mer (IFREMER) spanning the period from 2009 to 2019 was also used. This validated reanalysis dataset (combining scatterometer, radiometer, and atmospheric wind reanalysis) is available from Copernicus Marine Environment Monitoring Service (CMEMS).

### Douglas-Peucker line simplification algorithm

The DP line simplification algorithm, also known as the iterative end-point fit algorithm, is used to reduce the number of points representing a curve. The process approximates a given line by first connecting its terminal points and comparing the distance of each intermediate point with the tolerance parameter (ε). The intermediate point is retained if its distance from the straight line connecting the terminal points is greater than the ε value, else it is decimated. When an intermediate point is retained, the same process is iteratively applied to the line segments connecting that intermediate point and the previous terminal points. The final simplified line is such that the perpendicular distance of each point in the original unfiltered line and the simplified line segment is less than ε.

### Selection of coastal points

**Fig. 2:**
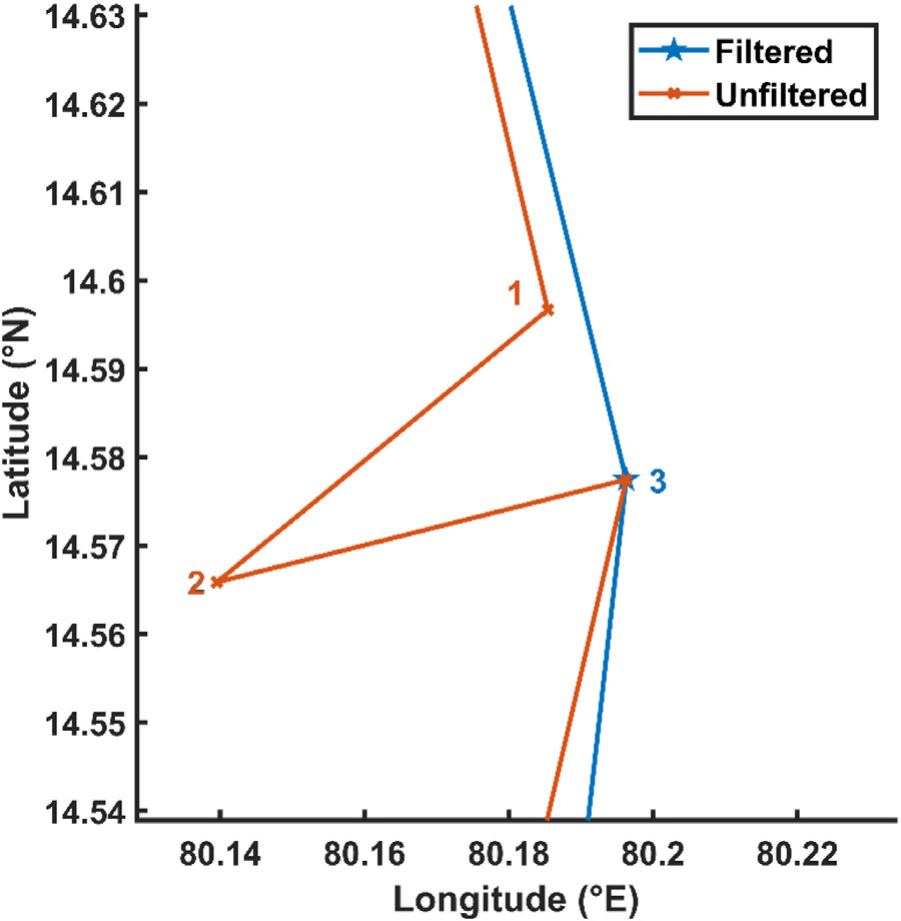
Example of coastline filtering.

First, the required polygon is selected from the GSHHS coastline file. In MATLAB, the 'BoundingBox' feature of the 'shaperead' function can be used to narrow down the search. Polygon properties can also be used to narrow down this search. Once the polygon is identified, the required coastline is extracted from this polygon by accepting only those points within given latitude-longitude bounds. These two steps (shaded in blue in Fig. 1) produce the dataset (the coordinates of the coastline), which serves as the input for the estimation process. Fig. 2 presents a simple demonstration of the third step. Following selection of the shoreline, two sets of points are deleted (decimated) from the dataset. The first set of points consist of internal coastline points associated with complicated structures such as bays, river outlets, or estuaries significantly smaller than the overall extent of the coastline (e.g., point 2 in Fig. 2). The hydrography within such isolated waters is often determined primarily through its connection with the open ocean and not local upwelling favourable forcing [15]. Now, the removal of these internal points could results in sets of points within close proximity to each other (e.g., the two points 1 and 3 in Fig. 2 near the inlet of an enclosed coastal feature). In such cases, one point from each group of such clustered neighbours is retained (point 3 in Fig. 2), while the others are rejected (point 1 in Fig. 2). The two preceding steps (filtering internal and clustered points, shaded in yellow in Fig. 1 are carried out manually. The resulting coastal points are the locations where the coastal angle and windstress values are then estimated. A set of 16 such coastal points along the AS and 23 such coastal points along the BoB are considered for demonstrating our method (Fig. 4). The list of the coastal points along with the corresponding coastal angle values are provided in the supplementary materials.

In the present analysis, internal and closely spaced coastal points were filtered out manually, though both of the steps involved (yellow box in Fig. 1) may be omitted to fully automate the ET computation process without affecting the ET estimates. Omitting these two steps could result in repeated ET values (for closely spaced coastal points) and minor gaps in ET data (at internal coastal points).

### Estimation of coastal angle

**Fig. 3:**
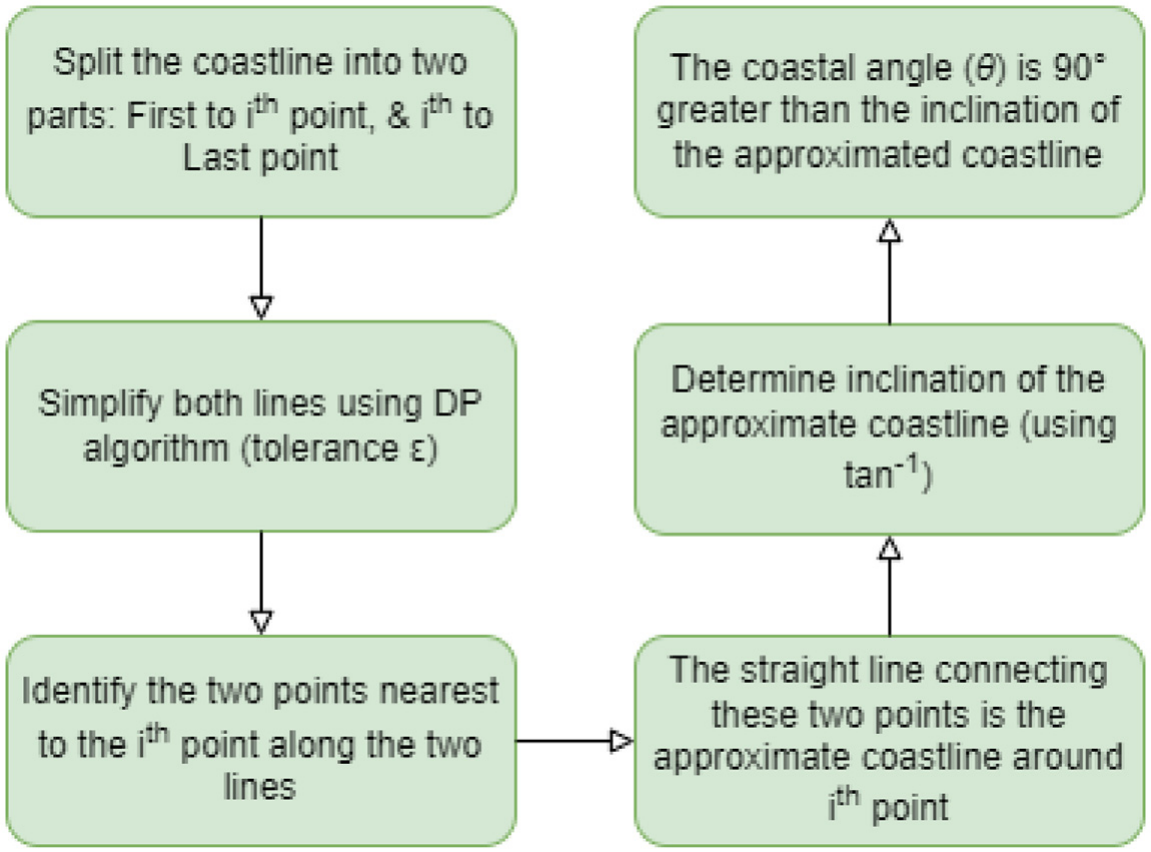
Coastal angle estimation process.

**Fig. 4:**
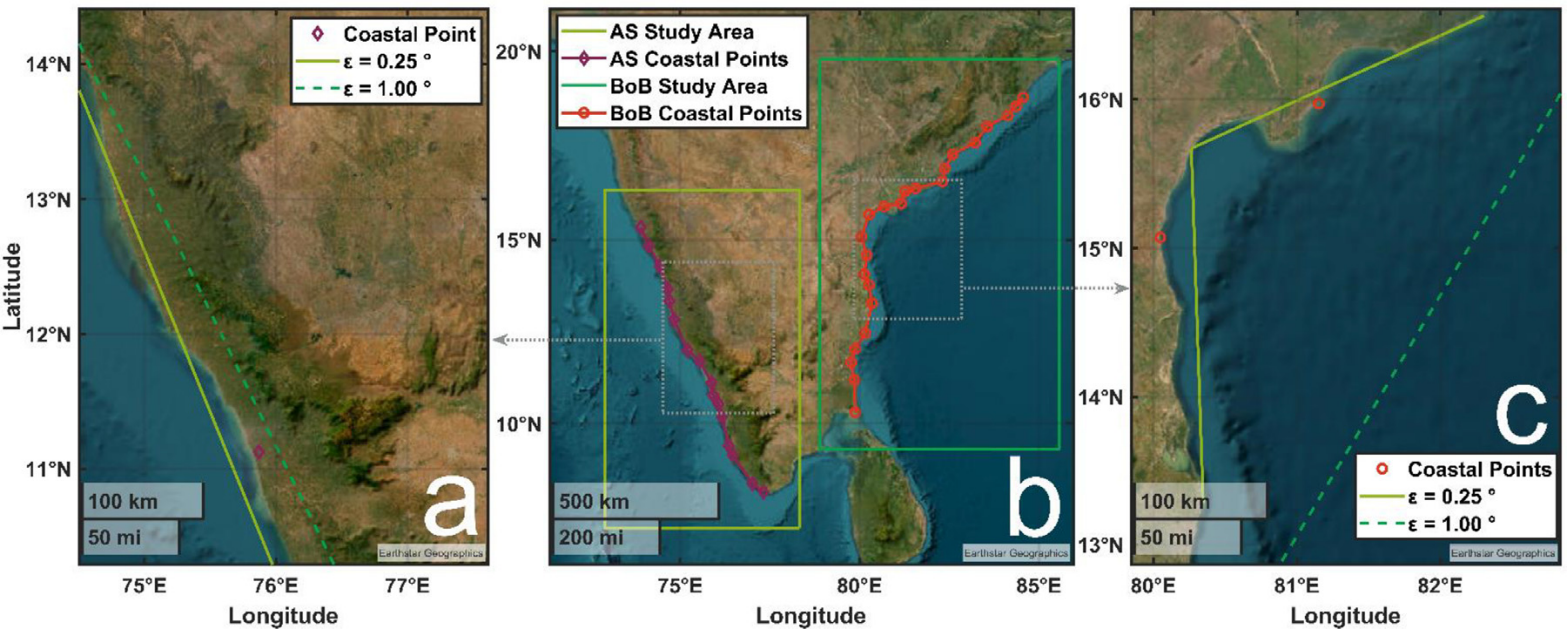
Schematic of the study area (b) with coastal points; and coastline simplification for (a) the western coast, and (c) the eastern coast of India.

The coastal angle is defined as the angle subtended by a normal to the coastline pointing seaward with the eastward direction in geometric convention (i.e. the angle is measured anti-clockwise with respect to the east). The coastal angle estimation process consists (outlined in Fig. 3) of six steps applied successively to each non-terminal filtered coastal point. When applied on the i^th^ coastal point, two lines are formed, one from the i^th^ point to each of the two terminal points (using the complete, unfiltered coastline data). These two lines are then simplified using the DP line simplification algorithm with a tolerance parameter value of ε. Next, we identify the closest neighbours of the ith point on the two simplified lines. The straight line connecting these two neighbouring points is the approximation of the coastline at that point. Its inclination can be estimated through the four-quadrant arctan of vertical and horizontal extents of this line. For very low values of ε, the nearest neighbours of the i^th^ point are the (*i* + 1)^th^ and (i-1)^th^ points (along the unfiltered coastline), while for very high values of ε, the nearest neighbours are the terminal points. Thus, low values of ε result in coastal angles computed from the central difference about each point (forward and backward differences are used for terminal points). In contrast, high ε values result in the whole coastline being treated as a single straight line (connecting the terminal points of the unfiltered coastline); this is illustrated for a few coastal points in Fig. 4a and 4c [16]. The coastal angle and ET determination method is applied to a section of the east coast of India along the AS (Fig. 4a), and a section of the west coast of India along the BoB (Fig. 4c). The dark green dashed line in Figs. 4a and c represents the case of high ε value where this single line is treated as the approximation of the entire shoreline. Additionally, the light green lines illustrate the case of low ε value, where the coastline is approximated using multiple line segments. The coastal angles are determined perpendicular to the approximate coastline, pointing seawards. Coastal angles corresponding to ε values ranging from 0.25° (the data grid spacing) to 2.5° were computed, with the later value resulting in a single-line approximation of the coastline.

### Alongshore windstress and Ekman transport

Once the coastal angle at each coastal point is determined, the surface windstress components at each coastal point are determined by interpolating the values from an observational windstress dataset using a single-pass of the distance-weighted Cressman window [17] with a critical radius of R. An R-value of 75 km (spanning roughly three grid points in each direction) was used in this analysis as the windstress data over one or two grid points close to the coastal points were sometimes unavailable. Next, the AWS and ET at each coastal point are computed following the methodology of ref [[5], [6], [7],^18^] (shaded red in Fig. 1). The alongshore windstress is computed as

$$\tau_{l}=\;-\left(\frac{lat}{abs\left(lat\right)}\right)\left(\tau_{x}\text{cos}\left(\theta -90^{\circ }\right)+\;\tau_{y}\text{sin}\left(\theta -90^{\circ }\right)\right)$$

$$M=\;\tau_{l}\;/\;\rho f$$

where $\tau_{l}$ is the AWS, θ is the coastal angle, M is the Ekman transport, ρ is the density of water, and f is the Coriolis parameter. lat represents the latitude in the interval [-90,90], while abs(lat) is its magnitude.

**Fig. 5:**
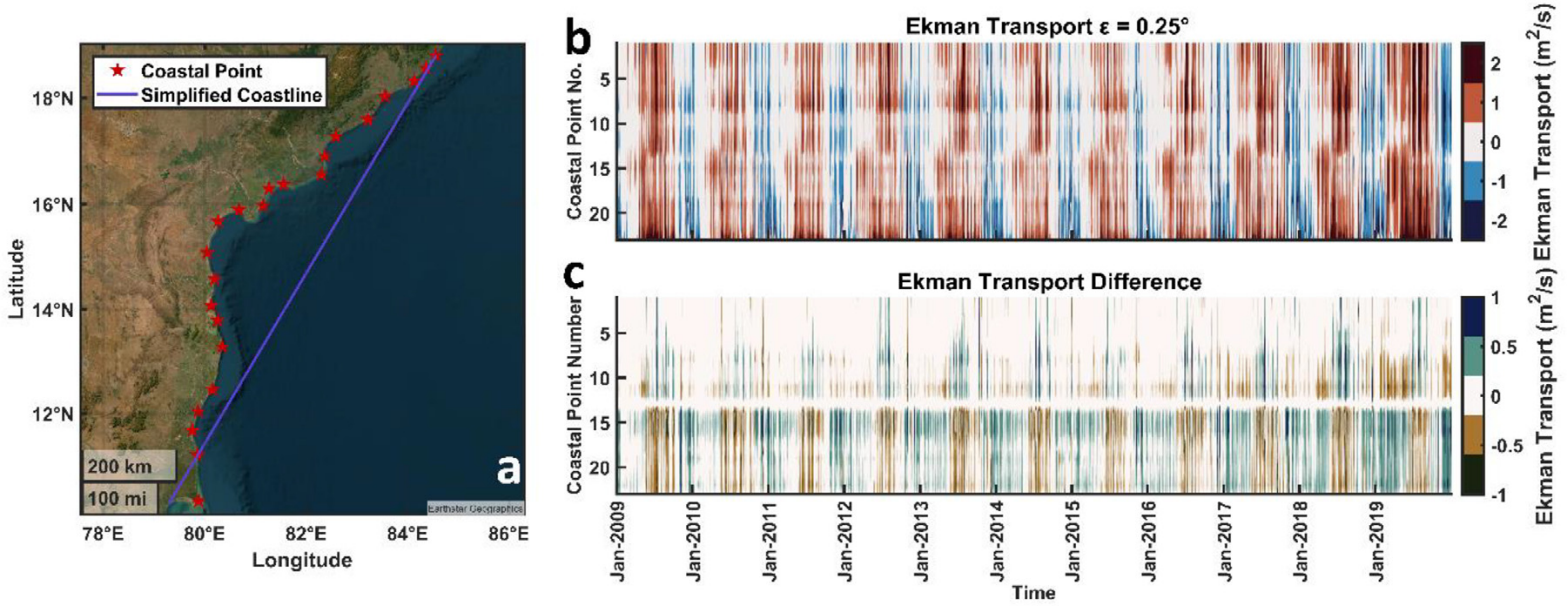
(a) Coastal points and the linear approximation of the eastern coastline of India (b) Time Series of ET along east coast of India computed with ε value of 0.25°, and (c) The time series of the difference between ET computed with ε values of 0.25° and 1.0°.

Twenty three coastal points along the east coast of India (Fig. 5a), the ET values computed through this method at each of these points (Fig. 5b) using 6-hourly scatterometer wind reanalysis data, and the difference between ET computed using ε values of 0.25° and 1.0° at each of these coastal points (Fig. 5c) are presented. To the south of point 13 (the minimum ET difference point), large ET differences persist throughout the year with an apparent seasonal oscillation. A similar seasonal oscillation is also observed in the ET values all along the east coast, with some noticeable alongshore variation in the periods of peak ET. The ET difference increases as the orientation of the coastline diverges from the single-line approximation of the coastline. This emphasized the need for accurate estimation of coastal angles in the determination of ET. An overtly coarse (high tolerance parameter or single-line approximation) produced a time varying error, that also varied greatly along the coast. In the northern part of the coast (north of the minimum error point “13″ in Fig. 5c), the error is in phase with the ET, while the errors are out of phase to its south. This highlighted the need to adopt a standard method of estimating coastal angles for ET computation, and such a method is presented here.

We next developed a time-averaged metric of the difference in ET, to quantify and analyze the variation of ET differences along the coast. This is indicative of the potential errors that may arise from the over-simplification of a coastline. The percentage difference in ET is computed as:

$$Percentage\;Difference=\;\frac{\Delta ET}{ET}\;\times \;100\%=\;\frac{mean\left(\;abs(ET_{\varepsilon =0.25}-\;ET_{\varepsilon =2.5}\;\right))}{mean\left(abs\left(\;ET_{\varepsilon =0.25}\;\right)\right)}\;\times \;100\%$$

**Fig. 6:**
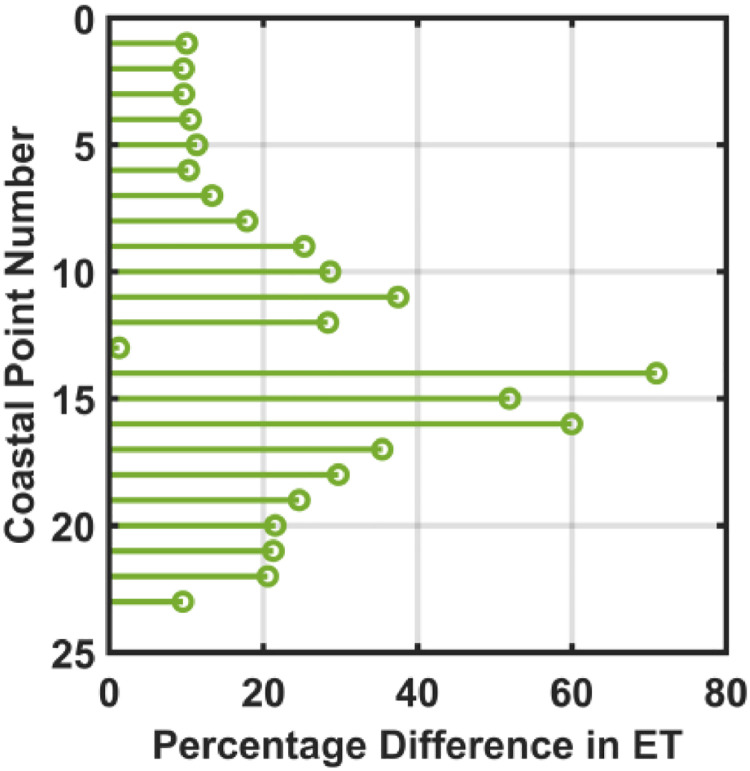
Mean percentage difference of ET at each coastal point.

The time mean of the percentage difference of ET at each coastal point over the period from 2009 to 2019 is presented in Fig. 6. The values revealed that the majority of coastal points exhibited errors greater than 20%, further demonstrating the necessity for systematic costal angle estimation.

**Fig. 7:**
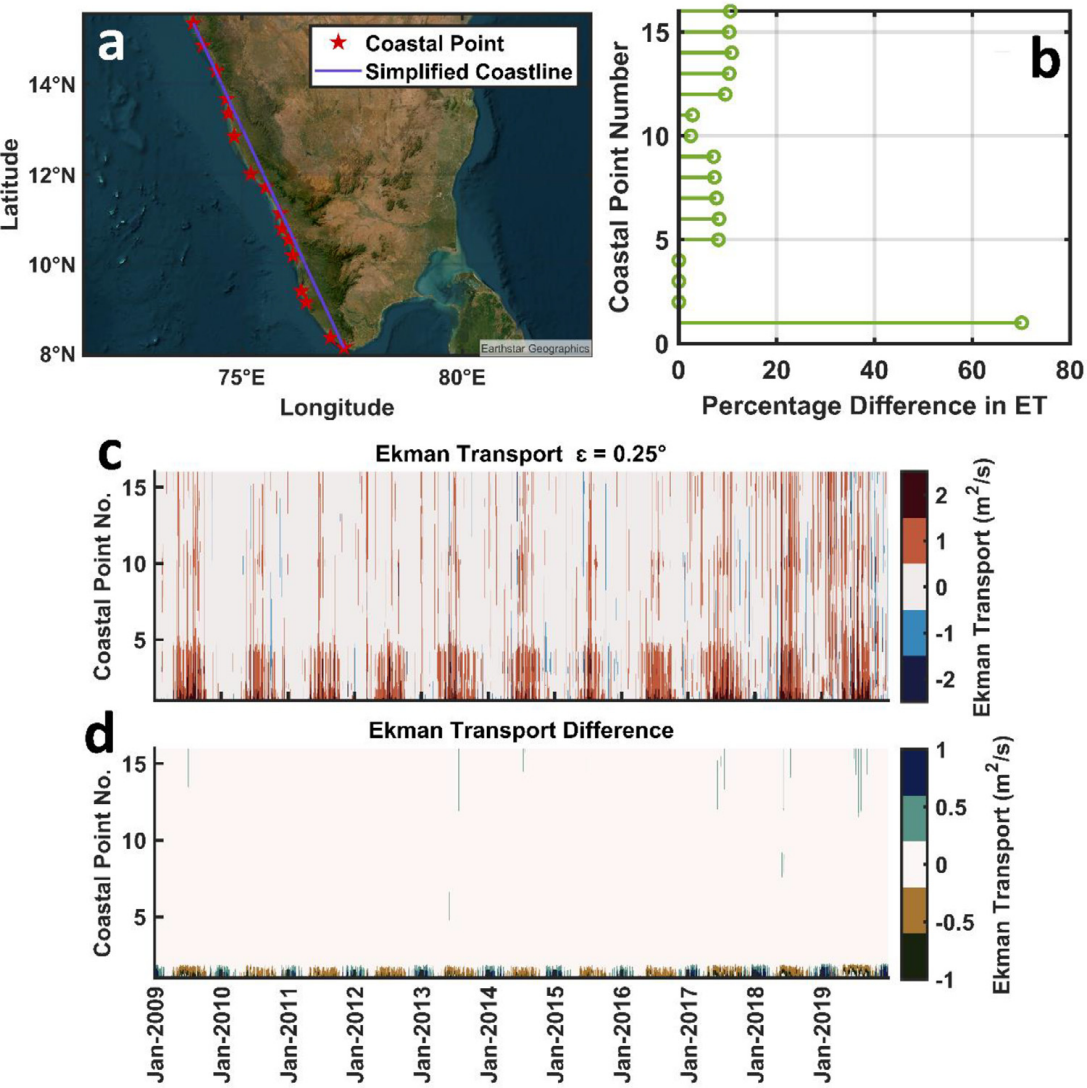
(a) Coastal points and the linear approximation of the western coastline of India, (b) Mean percentage difference of ET at each coastal point, and (c) ET along west coast of India computed with ε value of 0.25° d) the difference between ET computed with ε values of 0.25° and 1.0°.

The largest difference values (of ET al on the east coast) are observed south of point 13, with values ranging over 70% and between 10% and 40% to the north of the point (in Fig. 6). This demonstrates that using higher ε values (or single-line approximations) results in significant differences in the ET variations over coastlines with significant curvature (like the east coast of India). Compared to its east coast, the west coast of India is observed to be of a much lower curvature, and AWS is expected to be less sensitive to the choice of ε along such coastlines. To verify this, a plot identical to Fig. 6 was presented for the west coast of India in Fig. 7. It illustrated that a single-line approximation only resulted in *a* < 10% difference in ET values throughout the coast, except at the southernmost point (at the cape), where a sharp curvature in the coastline exists. Figs. 5 and 6 can be easily replicated for any coastline to judge whether a single-line approximation of the coastline is sufficient. Our previous analysis of the seasonal coastal upwelling along the east coast of India demonstrated that ET values computed with low ε agree more closely with other proxies of upwelling (such as an SST-based upwelling index) [16].

This method of determination of coastal angles and ET is applicable globally, irrespective of the level of complexity or curvature of the shoreline. Once the appropriate ε value is determined, the suitable level of simplification of the coastline necessary is determined systematically within this method, and the coastal angles are computed from it. A value of ε matching the grid spacing of the windstress data was found to be appropriate for the above computation [16]. While the original method and ε selection are based on the analysis of a basin scale coastal upwelling system, the method can also be extended to more localized upwelling systems.

The method can be utilized by researchers and operational agencies requiring near real-time estimates or forecasts of wind-based upwelling index (from scatterometers and numerical models). Such estimates have wide ranging application in identification of areas of ocean primary productivity, potential fishing zones, and oxygen depletion [19], [20], [21]. Additionally, it can be adopted for detailed analysis of small coastal upwelling systems which otherwise could not be considered due to issues with coastal angle computation owing to complex coastal geometries.

## Conclusions

Observational studies of coastal upwelling were found to be ambiguous with respect to the methodology of determining coastal angles, which are required for the computation of AWS [5], [6], [7], [8], [9], [10], [11]. In the case of coastlines without significant curvature, the coastline is often approximated using a single line segment, while coastlines with curvature necessitate multiple line segments. A review of the relevant literature [5], [6], [7], [8], [9], [10], [11] found no systematic method of either determining the number of line segments necessary to approximate a given coastline of arbitrary curvature, or no common approach of fitting line segments to coastlines. In this study, we have developed a parametric method of estimating coastal angles and demonstrated the computation of coastal upwelling-induced cross-shore ET on India's east and west coasts. This method allowed us to approximate the coastline as a single straight line or different straight lines at each of the coastal points by varying the line simplification tolerance parameter (ε) value. Further, the percentage difference in ET (as presented in Figs. 6 and 7b), allows a preliminary determination of the number of line segments (determined by the ε value and curvature of the coastline) necessary to approximate any given coastline. ET was estimated for a part of the east coast of India (a coastline with significant curvature), and it demonstrated that the choice of ε significantly affected ET values (with differences of up to 70%). However, for the west coast of India, a coastline without significant curvature, ET estimates are observed to be less sensitive to the choice of ε (with < 10% ET differences resulting from the use of the single-line approximation for all non-terminal coastal points). This systematic ET computation method can be extended to any coastal upwelling system, for which the tolerance parameter value may be chosen based on the resolution of available windstress data, the coastline curvature, and the spatial scale of interest. Such a method can enable globally consistent estimates of ETs and therefore allow a more systematic and reliable computation of AWS over different geographical regions.

## Background

Coastal upwelling is one of the most significant drivers of ocean productivity. The major eastern boundary coastal upwelling systems comprising only 1% of the ocean surface area contribute to over 20% of the productivity of the global oceans [1]. It is primarily driven by alongshore Windstress (AWS), which arises from ocean surface winds aligned parallel to the coast, with the coast to the left (right) of the wind in the northern (southern) hemisphere. Such a positive AWS produces a frictionally driven cross-shore surface flow (Ekman transport) directed away from the coast. This, in turn, results in the upward flow of deeper waters to balance the coastal divergence resulting from the offshore Ekman transport. This upward flow of deeper waters, referred to as "coastal upwelling", transports cold nutrient-rich waters into the near-surface euphotic layer, resulting in phytoplankton blooms that form the base of the marine food web and support larger marine organisms [2]. Thus, coastal upwelling is one of the most significant air-sea interaction processes that determine the physical and bio-chemical hydrography of coastal waters [3], [4].

The AWS is computed based on surface windstress observations (typically from satellite-borne scatterometers), and coastal angles are determined through various methods. There are no well-established standard methods of estimation of coastal angles, and the literature largely remains ambiguous in this regard. Coastlines without significant curvature (e.g. the west coast of India) are usually approximated by a single straight line [5], [6], [7], [8], [9] for the ease of computation of coastal angles. When it is impossible to adopt this simplifying approach, different line segments parallel to the coastline are fitted at different points along the coast [10], [11]. No standard method for this is found in the literature. In particular, the choice between single-line and multi-line approximations of the coast is also subjective, with examples of the use of both available for the same coastlines [5,11]. The level of simplification (i.e., reduction in the number of points used to represent the polygon) of the coastline plays a crucial role in determining the coastal angle when different line segments are fitted to different points along the coast. This can introduce significant ambiguities in the computation of AWS. This publication attempts to develop a parametric method of computation of coastal angles, such that coastal angles corresponding to both the single-line and multi-line approximations of the coastline can be computed systematically by varying the parameter value.

## Supplementary material

The coastline dataset for the east and west coast of India, including the coastal angle estimates are available in the supplementary materials.

## CRediT authorship contribution statement

**Sthitapragya Ray:** Conceptualization, Methodology, Formal analysis, Writing – original draft. **Debadatta Swain:** Supervision, Writing – review & editing.

## Declaration of Competing Interest

The authors declare that they have no known competing financial interests or personal relationships that could have appeared to influence the work reported in this paper.

## Data Availability

Data will be made available on request.

## Appendix Supplementary materials

Image, application 1

## References

[1] Botsford L.W., Lawrence C.A., Dever E.P., Hastings A., Largier J. (2006). Effects of variable winds on biological productivity on continental shelves in coastal upwelling systems. Deep Sea Res. II Top. Stud. Oceanogr..

[2] Bakun A., Black B.A., Bograd S.J., García-Reyes M., Miller A.J., Rykaczewski R.R., Sydeman W.J. (2015). Anticipated effects of climate change on coastal upwelling ecosystems. Curr. Clim. Change Rep..

[3] Wang B., Wang X. (2007). Chemical hydrography of coastal upwelling in the East China Sea. Chin. J. Ocean. Limnol..

[4] Peterson W.T., Keister J.E., Feinberg L.R. (2002). The effects of the 1997–99 El Niño/La Niña events on hydrography and zooplankton off the central Oregon coast. Prog. Oceanogr..

[5] Jayaram C., Chacko N., Joseph K.A., Balchand A.N. (2010). Interannual variability of upwelling indices in the Southeastern Arabian Sea: a satellite based study. Ocean Sci. J..

[6] Jayaram C., Kumar P.K.D. (2018). Spatio-temporal variability of upwelling along the southwest coast of India based on satellite observations. Cont. Shelf Res..

[7] Jayaram C., Jose F. (2022). Relative dominance of wind stress curl and Ekman transport on coastal upwelling during summer monsoon in the southeastern Arabian Sea. Cont. Shelf Res..

[8] Shetye S.R. (1984). Seasonal variability of the temperature field off the south-west coast of India. Proc. Indian Acad. Sci. Earth Planet Sci..

[9] Santos F., Gomez-Gesteira M., deCastro M., Alvarez I. (2012). Differences in coastal and oceanic SST trends due to the strengthening of coastal upwelling along the Benguela current system. Cont. Shelf Res..

[10] Ganguly D., Raman M. (2021). Proceedings of the IEEE International India Geoscience and Remote Sensing Symposium (InGARSS).

[11] Shah P., Sajeev R., Gopika N. (2015). Study of upwelling along the West Coast of India—a climatological approach. J. Coast. Res..

[12] Wessel P., Smith W.H.F. (1996). A global, self-consistent, hierarchical, high-resolution shoreline database. J. Geophys. Res. Solid Earth.

[13] E.A. Soluri, V.A. Woodson, World vector shoreline, Int. Hydrogr. Rev. 67(1) (1990) 27-35, https://journals.lib.unb.ca/index.php/ihr/article/view/23315 Accessed December 28, 2022.

[14] Douglas D.H., Peucker T.K. (1973). Algorithms for the reduction of the number of points required to represent a digitized line or its caricature. Cartographica.

[15] Roegner G.C., Needoba J.A., Baptista A.M. (2011). Coastal upwelling supplies oxygen-depleted water to the Columbia River Estuary. PLoS One.

[16] Ray S., Swain D., Ali M.M., Bourassa M.A. (2022). Coastal upwelling in the Western Bay of Bengal: role of local and remote windstress. Remote Sens..

[17] Cressman G.P. (1959). An operational objective analysis system. Mon. Weather Rev..

[18] Varela R., Álvarez I., Santos F., deCastro M., Gómez-Gesteira M. (2015). Has upwelling strengthened along worldwide coasts over 1982-2010?. Sci. Rep..

[19] Carr M.E. (2001). Estimation of potential productivity in eastern boundary currents using remote sensing. Deep Sea Res. Part II Top. Stud. Oceanogr..

[20] Pitcher G.C., Aguirre-Velarde A., Breitburg D., Cardich J., Carstensen J., Conley D.J., Zhu Z.Y. (2021). System controls of coastal and open ocean oxygen depletion. Prog. Oceanogr..

[21] Ramajo L., Valladares M., Astudillo O., Fernández C., Rodríguez-Navarro A.B., Watt-Arévalo P., Tapia C. (2020). Upwelling intensity modulates the fitness and physiological performance of coastal species: implications for the aquaculture of the scallop Argopecten purpuratus in the Humboldt current system. Sci. Total Environ..

[22] Greene C.A., Thirumalai K., Kearney K.A., Delgado J.M., Schwanghart W., Wolfenbarger N.S., Thyng K.M., Gwyther D.E., Gardner A.S., Blankenship D.D. (2019). The climate data toolbox for MATLAB. Geochem. Geophys. Geosyst..

[23] W. Schwanghart Line simplification. MATLAB Central File Exchange. 2021. Available online: https://www.mathworks.com/matlabcentral/fileexchange/21132-line-simplification (accessed on 11 July 2022).

