# Supplementary material for: A systematic method of estimation of alongshore windstress and Ekman transport associated with coastal upwelling
Source: MethodsX. 2023 Apr 15;10:102186. doi: 10.1016/j.mex.2023.102186 (PMC10148182; doi:10.1016/j.mex.2023.102186)
Supplement: Supplementary file 1 [file mmc1.docx]

**Supplementary Materials**

**Table 1:** Unfiltered Coastline for the East Coast of India

| Point Number | Latitude (°N) | Longitude (°E) |
| --- | --- | --- |
| 1 | 18.79 | 84.56 |
| 2 | 18.53 | 84.31 |
| 3 | 18.55 | 84.36 |
| 4 | 18.35 | 84.10 |
| 5 | 18.31 | 84.13 |
| 6 | 18.03 | 83.56 |
| 7 | 17.59 | 83.21 |
| 8 | 17.28 | 82.59 |
| 9 | 17.04 | 82.31 |
| 10 | 16.88 | 82.25 |
| 11 | 16.84 | 82.35 |
| 12 | 16.99 | 82.33 |
| 13 | 16.91 | 82.37 |
| 14 | 16.56 | 82.30 |
| 15 | 16.31 | 81.72 |
| 16 | 16.36 | 81.72 |
| 17 | 16.31 | 81.71 |
| 18 | 16.37 | 81.55 |
| 19 | 16.29 | 81.27 |
| 20 | 15.97 | 81.15 |
| 21 | 15.71 | 80.94 |
| 22 | 15.76 | 80.90 |
| 23 | 15.71 | 80.92 |
| 24 | 15.70 | 80.83 |
| 25 | 15.84 | 80.81 |
| 26 | 15.89 | 80.68 |
| 27 | 15.67 | 80.26 |
| 28 | 15.07 | 80.05 |
| 29 | 14.60 | 80.19 |
| 30 | 14.57 | 80.14 |
| 31 | 14.58 | 80.20 |
| 32 | 14.25 | 80.10 |
| 33 | 14.07 | 80.13 |
| 34 | 13.78 | 80.26 |
| 35 | 13.28 | 80.35 |
| 36 | 12.46 | 80.16 |
| 37 | 12.04 | 79.87 |
| 38 | 11.67 | 79.76 |
| 39 | 11.36 | 79.83 |
| 40 | 11.38 | 79.76 |
| 41 | 11.36 | 79.84 |
| 42 | 11.20 | 79.86 |
| 43 | 10.31 | 79.88 |
| 44 | 10.27 | 79.79 |
| 45 | 10.37 | 79.64 |
| 46 | 10.26 | 79.29 |

**Table 2:** Unfiltered Coastline for the West Coast of India

| Point Number | Latitude (°N) | Longitude (°E) |
| --- | --- | --- |
| 1 | 8.07 | 77.54 |
| 2 | 8.12 | 77.32 |
| 3 | 8.35 | 77.01 |
| 4 | 8.90 | 76.55 |
| 5 | 8.99 | 76.67 |
| 6 | 8.93 | 76.54 |
| 7 | 9.17 | 76.50 |
| 8 | 9.25 | 76.43 |
| 9 | 9.14 | 76.46 |
| 10 | 9.39 | 76.35 |
| 11 | 10.03 | 76.27 |
| 12 | 9.97 | 76.24 |
| 13 | 10.20 | 76.21 |
| 14 | 10.19 | 76.16 |
| 15 | 10.54 | 76.07 |
| 16 | 10.78 | 75.92 |
| 17 | 10.82 | 75.96 |
| 18 | 10.78 | 75.91 |
| 19 | 11.12 | 75.87 |
| 20 | 11.48 | 75.62 |
| 21 | 11.71 | 75.54 |
| 22 | 11.97 | 75.31 |
| 23 | 12.00 | 75.20 |
| 24 | 12.84 | 74.83 |
| 25 | 13.34 | 74.70 |
| 26 | 13.63 | 74.67 |
| 27 | 13.64 | 74.72 |
| 28 | 13.66 | 74.65 |
| 29 | 14.28 | 74.43 |
| 30 | 14.24 | 74.52 |
| 31 | 14.29 | 74.42 |
| 32 | 14.71 | 74.28 |
| 33 | 14.77 | 74.12 |
| 34 | 14.86 | 74.17 |
| 35 | 14.84 | 74.12 |
| 36 | 15.08 | 73.91 |
| 37 | 15.16 | 73.96 |
| 38 | 15.33 | 73.90 |
| 39 | 15.41 | 73.78 |
| 40 | 15.43 | 73.89 |
| 41 | 15.46 | 73.79 |
| 42 | 15.50 | 73.83 |
| 43 | 15.49 | 73.77 |
| 44 | 15.59 | 73.73 |
| 45 | 15.65 | 73.79 |
| 46 | 15.61 | 73.73 |
| 47 | 15.87 | 73.63 |

**Table 3:** Filtered Coastline for the East Coast of India with Coastal Angles

| Point Number | Latitude (°N) | Longitude (°E) | Coastal Angle (°) | | | |  |
| --- | --- | --- | --- | --- | --- | --- | --- |
|  |  |  | ε = 0.25° | ε = 0.5° | ε = 1.0° | ε = 2.5° | |
| 1 | 18.79 | 84.56 | 307.88 | 305.99 | 305.99 | 328.32 | |
| 2 | 18.55 | 84.36 | 307.88 | 305.99 | 305.99 | 328.32 | |
| 3 | 18.31 | 84.13 | 307.88 | 305.99 | 305.99 | 328.32 | |
| 4 | 18.03 | 83.56 | 307.88 | 305.99 | 305.99 | 328.32 | |
| 5 | 17.59 | 83.21 | 307.88 | 305.99 | 305.99 | 328.32 | |
| 6 | 17.28 | 82.59 | 314.70 | 305.99 | 305.99 | 328.32 | |
| 7 | 16.91 | 82.37 | 310.40 | 305.99 | 305.99 | 328.32 | |
| 8 | 16.56 | 82.30 | 303.79 | 305.99 | 305.99 | 328.32 | |
| 9 | 16.37 | 81.55 | 301.88 | 305.99 | 328.32 | 328.32 | |
| 10 | 16.29 | 81.27 | 301.88 | 309.49 | 328.32 | 328.32 | |
| 11 | 15.97 | 81.15 | 293.51 | 309.49 | 328.32 | 328.32 | |
| 12 | 15.89 | 80.68 | 305.51 | 309.49 | 328.32 | 328.32 | |
| 13 | 15.67 | 80.26 | 329.14 | 331.12 | 328.32 | 328.32 | |
| 14 | 15.07 | 80.05 | 361.98 | 331.12 | 328.32 | 328.32 | |
| 15 | 14.58 | 80.20 | 355.91 | 355.91 | 328.32 | 328.32 | |
| 16 | 14.07 | 80.13 | 361.98 | 355.91 | 328.32 | 328.32 | |
| 17 | 13.78 | 80.26 | 352.80 | 355.91 | 349.84 | 328.32 | |
| 18 | 13.28 | 80.35 | 352.80 | 355.91 | 349.84 | 328.32 | |
| 19 | 12.46 | 80.16 | 352.80 | 355.91 | 349.84 | 328.32 | |
| 20 | 12.04 | 79.87 | 351.10 | 355.91 | 349.84 | 328.32 | |
| 21 | 11.67 | 79.76 | 351.10 | 355.91 | 349.84 | 328.32 | |
| 22 | 11.20 | 79.86 | 351.10 | 349.84 | 349.84 | 328.32 | |
| 23 | 10.31 | 79.88 | 341.78 | 349.84 | 349.84 | 328.32 | |

**Table 4:** Filtered Coastline for the West Coast of India with Coastal Angles

| Point Number | Latitude (°N) | Longitude (°E) | Coastal Angle (°) | | | |
| --- | --- | --- | --- | --- | --- | --- |
|  |  |  | ε = 0.25° | ε = 0.5° | ε = 1.0° | ε = 2.5° |
| 1 | 8.12 | 77.32 | 230.22 | 206.62 | 206.62 | 206.62 |
| 2 | 8.35 | 77.01 | 206.62 | 206.62 | 206.62 | 206.62 |
| 3 | 9.14 | 76.46 | 206.62 | 206.62 | 206.62 | 206.62 |
| 4 | 9.39 | 76.35 | 206.62 | 206.62 | 206.62 | 206.62 |
| 5 | 10.19 | 76.16 | 202.71 | 206.62 | 206.62 | 206.62 |
| 6 | 10.54 | 76.07 | 202.71 | 206.62 | 206.62 | 206.62 |
| 7 | 10.78 | 75.91 | 202.71 | 206.62 | 206.62 | 206.62 |
| 8 | 11.12 | 75.87 | 202.71 | 206.62 | 206.62 | 206.62 |
| 9 | 11.71 | 75.54 | 202.71 | 206.62 | 206.62 | 206.62 |
| 10 | 12.00 | 75.20 | 205.26 | 206.62 | 206.62 | 206.62 |
| 11 | 12.84 | 74.83 | 205.26 | 206.62 | 206.62 | 206.62 |
| 12 | 13.34 | 74.70 | 202.71 | 206.62 | 206.62 | 206.62 |
| 13 | 13.66 | 74.65 | 202.71 | 202.71 | 206.62 | 206.62 |
| 14 | 14.29 | 74.42 | 202.71 | 202.71 | 206.62 | 206.62 |
| 15 | 14.84 | 74.12 | 202.71 | 202.71 | 206.62 | 206.62 |
| 16 | 15.33 | 73.90 | 202.71 | 202.71 | 206.62 | 206.62 |
